# CONSEQUENCES OF ECONOMIC HARDSHIP AND SELF-RATED HEALTH AMONG OLDER ADULTS

**DOI:** 10.1093/geroni/igz038.455

**Published:** 2019-11-08

**Authors:** Yang Li, Jan Mutchler

**Affiliations:** University of Massachusetts Boston, Boston, Massachusetts, United States

## Abstract

Adequate economic resources ensure that older adults’ basic needs are met and facilitate a healthier lifestyle. Hardship signals unfulfilled needs experienced by individuals lacking adequate economic resources. Despite well-documented associations between indicators of hardship and self-rated health, little is known about whether hardship has the same impact on self-rated health across age groups. The purpose of this study was to investigate the association between hardship and self-rated health among older adults and determine whether this association differed by age. Employing data from the 2014 Survey of Income and Program Participation, we conducted logistic regression analysis to examine the association between hardship and self-rated health among adults age 55 and older in the United States, and the moderating effect of age on this relationship. Analyses were weighted using replicate weights provided by the survey. Indicators of hardship were dichotomized (1 = experienced hardship, 0 = no hardship). Analyses indicated that individuals who were unable to pay utility bills, unable to pay rent or mortgage, or who experienced food insecurity had lower odds of reporting good/very good/excellent health relative to those not experiencing these hardships. The association between hardship and self-rated health was shown to be less substantial among the oldest cohort (age 75 and older) relative to younger adults. Hardship is directly relevant to health outcomes as it represents the consequence of unfulfilled needs experienced by individuals lacking adequate economic resources. This study contributes to our understanding of the role of age in the association between hardship and self-rated health.

